# PRESERFLO MicroShunt implantation versus trabeculectomy for primary open-angle glaucoma: a two-year follow-up study

**DOI:** 10.1186/s40662-023-00369-8

**Published:** 2023-12-21

**Authors:** Pascal Aurel Gubser, Valentin Pfeiffer, Simon Hug, Xiao Shang, Joel-Benjamin Lincke, Nathanael Urs Häner, Martin S. Zinkernagel, Jan Darius Unterlauft

**Affiliations:** Department of Ophthalmology, Inselspital, Bern University Hospital, University of Bern, Bern, Switzerland

**Keywords:** Glaucoma, Glaucoma surgery, Intraocular pressure, Optical coherence tomography

## Abstract

**Background:**

To compare the intermediate-term efficacy of PRESERFLO (PF) MicroShunt implantation with trabeculectomy (TE) in patients with primary open-angle glaucoma, focusing on longitudinal changes of functional and structural parameters.

**Methods:**

This retrospective comparative study included 104 eyes of 104 patients who underwent TE and 83 eyes of 83 patients that underwent PF implantation between January 2019 and December 2020, with a minimum follow-up of two years. Baseline and postoperative intraocular pressure (IOP), number of IOP-lowering medications, visual field mean defect (MD) and peripapillary retinal nerve fibre layer (RNFL) thickness measured using optical coherence tomography were assessed and compared between groups.

**Results:**

Baseline characteristics (age, sex, IOP, number of IOP-lowering medications, MD, RNFL thickness) were comparable between the two groups (all *P* > 0.05). During the two-year of follow-up, mean IOP decreased from 24.09 ± 1.15 mmHg and 21.67 ± 0.77 mmHg to 11.37 ± 1.13 mmHg (*P* < 0.001) and 15.50 ± 1.54 mmHg (*P* = 0.028), and the mean number of IOP-lowering medications decreased from 3.25 ± 0.14 and 3.51 ± 0.14 to 0.53 ± 0.14 (*P* < 0.001) and 1.06 ± 0.43 (*P* < 0.001) in the TE and PF groups, respectively. MD remained stable [− 11.54 ± 0.93 dB and − 11.17 ± 1.66 to − 10.67 ± 0.91 dB (*P* = 0.226) and − 10.40 ± 4.75 dB (*P* = 0.628) in the TE and PF groups, respectively] but RNFL thickness decreased continuously during follow-up [62.79 ± 1.94 µm and 62.62 ± 2.05 µm to 57.41 ± 1.81 µm (*P* < 0.001) and 60.22 ± 1.98 µm (*P* = 0.182) in the TE and PF groups, respectively].

**Conclusion:**

PF implantation is comparably effective in the intermediate term in lowering IOP and reducing the use of IOP-lowering medications over a two-year follow-up period. Although visual field defects were stable, RNFL continued to decrease during postoperative follow-up.

## Background

Glaucoma is a heterogeneous group of optic neuropathies that are characterized by the development of typical visual field defects and optic nerve (head) atrophy due to apoptotic demise of retinal ganglion cells (RGCs) [1–4]. Lowering intraocular pressure (IOP) remains the only known therapeutic option to slow or at best halt further disease progression [5–8]. Lowering IOP can be achieved by means of medical treatment, application of lasers (e.g., selective laser trabeculoplasty, laser iridotomy, cyclophotocoagulation) and surgical interventions (with and without the use of implants) [9, 10]. Trabeculectomy (TE) is the most performed glaucoma surgery because of its efficiency in lowering IOP and medication burden. Its efficacy has already been demonstrated in several long-term multicenter clinical trials [11–14]. However, TE has several disadvantages including the risk for serious vision-threatening adverse events requiring postoperative interventions, long postoperative convalescence times, and substantial surgical trauma [15–17].

Therefore, less invasive alternative surgical techniques with lower risks for vision-threatening adverse events were developed. In recent years, a few techniques have been developed that are collectively referred to as minimally invasive glaucoma surgery (MIGS) [18, 19]. One of these new techniques is the PRESERFLO (PF) MicroShunt, which functions through subconjunctival drainage of aqueous humour from the anterior chamber [20, 21]. TE and PF have a very similar mechanism of action through subconjunctival/sub-Tenon's filtration of aqueous humour. Several studies have already demonstrated the potential of PF to be a valuable addition to TE as it is able to decrease IOP and to reduce the number of IOP-lowering medications in the short and medium term after implantation [22–27].

The current study aimed to compare the IOP-lowering efficacy of TE and PF in the medium term after surgery. Secondary outcome measures were progression of visual field mean defect (MD) and peripapillary retinal nerve fibre layer (RNFL) thickness measured using optical coherence tomography (OCT).

## Methods

This retrospective comparative study was approved by the Ethics Commission of the Canton of Bern (BASEC-ID:ID 2022-01046). The need for participation consent was waived due to the retrospective nature of this study. Written informed consent for all surgical procedures performed was obtained from each patient prior to the scheduled surgery. The study protocol was in accordance with the tenets of the Declaration of Helsinki.

### Eligibility criteria

The records of primary open-angle glaucoma (POAG) patients who underwent TE or PF implantation between January 2019 and December 2020 at the Department of Ophthalmology, Inselspital, Bern University Hospital, Bern, Switzerland, were retrospectively reviewed. For POAG diagnosis, the presence of typical glaucomatous optic disc cupping with localized or generalized neuroretinal rim loss was mandatory. Additionally, a history of untreated IOP equal or above 21 mmHg had to be present. Other than glaucomatous optic disc cupping and elevated IOP, visual field defects did not have to be present (pre-perimetric POAG). When visual field defects were present, their localization had to fit in with the present glaucomatous nerve fibre loss. Cases of ocular hypertension without glaucomatous optic disc cupping or RNFL loss and absence of visual field defects were not included into this study.

Indication for glaucoma surgery was disease progression or above-target IOP on maximum tolerable medical therapy or the inability to escalate medical therapy further due to existing allergies or other medical conditions. Disease progression was defined as a worsening of the visual field MD on three consecutive tests with an increase of at least − 2.00 dB within one year. To be included in this study, patients had to be older than 40 years. Data including IOP, number of IOP-lowering medications, best-corrected visual acuity (BCVA), standard automated perimetry results, and peripapillary OCT measurements had to be available. Patients were excluded if they had any other coexisting ocular diseases that could interfere with IOP measurements, visual field testing, or OCT examination. All eyes suffering from any other glaucoma entity apart from POAG were excluded. Normal-tension glaucoma cases were also not included, since these were usually treated with TE and not PF, because of the need for a more pronounced IOP decrease. If both eyes were eligible, one eye was selected randomly.

### Surgical procedures

The decision to perform incisional glaucoma surgery was usually made at the discretion of the treating physician during outpatient examination. Surgical techniques followed previously published standard techniques for TE and PF MicroShunt implantation [28, 29].

TE: Performed using a fornix-based technique, application of 0.2 mg/mL mitomycin C (MMC) with sponges for 2 min followed by meticulous rinsing with balanced salt solution (BSS). A 4.00 × 4.00 mm scleral flap was created underneath which the anterior chamber was entered with a diamond knife. The scleral flap was then reapproximated with two to five non-absorbable 10/0 single-button sutures. The number of flap sutures was dependent on the resulting intraoperative IOP and the visible protrusion of aqueous humour underneath the scleral flap. Tenon’s capsule and conjunctiva were then reapproximated with two to four absorbable single button sutures and one to two non-absorbable mattress sutures to ensure water tightness at the limbus.

PF: For implantation of the PF MicroShunt, a fornix-based conjunctival flap was created in the superior-nasal or superior-temporal quadrant. The episclera and sclera were then treated with three sponges soaked in 0.2 mg/mL MMC for 2 min, placed away from the corneal limbus and then meticulously rinsed with BSS. Light wet-field cautery was applied to the episclera/sclera. A scleral tunnel was created starting 3.00 mm behind the limbus using a 1.00 mm paracentesis and a 25G angled needle, entering the anterior chamber at the chamber angle approximately at the trabecular meshwork. The implant was then inserted into the created scleral tunnel, with the device’s fin fitting tightly into the tunnel’s ostium. Correct positioning was visually verified with the tip of the device positioned bevel-up in the anterior chamber. Patency was verified by visible fistulation of aqueous humour at the end of the device. The Tenon’s capsule and conjunctiva were then reapproximated to the limbus and fixed with four absorbable 10/0 single-button sutures and one non-absorbable 10/0 mattress suture with the knots buried in the tissue.

Neither TE nor PF implantation were combined with phacoemulsification and intraocular lens implantation in the included cases. During the postoperative course, laser suture lysis (TE) or subconjunctival injection of 5-fluorouracil (5-FU) (TE and PF) were performed as needed, depending on the bleb morphology during slit-lamp examination or IOP measurements, usually only during the first three to six months after surgery. The postoperative medical treatment regimen was similar in both TE and PF groups. Immediately after surgery, medical treatment was started with topical antibiotics (tobramycin, four times per day for the first two weeks), topical steroids (prednisolone acetate, four times per day for the first two weeks, then tapered over the next eight weeks according to clinical assessment), and cycloplegics (atropine 1%, twice per day for one week). Needling of the subconjunctival/sub-Tenon’s space and postoperative injection of 5-FU (0.1 mL at a concentration of 50.0 mg/mL) was considered a regular part of postoperative treatment in both groups. The decision for needling and/or 5-FU injection was based on clinical assessment of the conjunctival filtration zone during slit lamp examination and IOP measurements. The decision to perform 5-FU injections or needling procedures was made at the clinical discretion of the treating physician but did not follow a set decision path. However, clinical decision making considered the prominence, vascularity, and the overall clinical appearance of the resulting conjunctival filtration zone and IOP. Needling procedure was performed under the operating microscope under sterile conditions with the patient in the supine position. 5-FU injection was performed with the patient seated in the chin-up position with eyes gazing downwards.

### Data collection and analysis

Demographic data including age, sex, and laterality of the operated eye were collected preoperatively. Baseline (one day before surgery) and follow-up visits (1, 3, 6, 12 and 24 months after surgery) data including IOP (Goldmann applanation tonometry, Haag-Streit, Köniz, Switzerland), the number of IOP-lowering medications, and BCVA were also collected. Peripapillary RNFL thickness measured using spectral domain OCT (Spectralis OCT, Heidelberg Engineering, Heidelberg, Germany) was evaluated at baseline and 3, 6, 12 and 24 months after surgery. Visual field MD assessed by standard automated perimetry (Octopus, Haag-Streit, Köniz, Switzerland) was evaluated at baseline and 6, 12 and 24 months after surgery.

Electronic data were collected using Excel (Microsoft, Redmond, Washington, USA). Statistical analysis was performed using SPSS (IBM, version 24.0; Chicago, Illinois, USA). Continuous variables were described as mean and standard error as well as median and interquartile range. Categorical variables were described as frequencies. Non-parametric Wilcoxon test was utilized for paired, within-group comparisons, non-parametric Kruskal–Wallis test was used for unpaired group comparisons (> two groups) and Mann–Whitney U test was used for unpaired between-group comparisons (two groups). Comparisons between groups concerning occurrence and frequency of postoperative interventions and complications was performed using the Fisher’s Exact test. Post-hoc Bonferroni correction was used for multiple testing when necessary. Additionally, the cumulative probability of success was assessed using Kaplan–Meier survival curves. A *P* value of less than 0.05 was considered as statistically significant.

## Results

104 eyes of 104 patients that underwent TE and 83 eyes of 83 patients that underwent PF implantation were enrolled (Table 1). Baseline demographics and ocular characteristics are shown in Table 1. In the TE group, mean patient age was 70.00 ± 12.70 years (range: 42 to 85 years) and the right eye was operated in 51 cases (49.03%). 64 patients were female, and 40 patients were male. Seven eyes (6.73%) had prior incisional glaucoma surgery (deep sclerectomy in all cases). In the PF implantation group, 44 were female and 39 were male with a mean age of 75.69 ± 10.84 years (range: 57 to 96 years). In 37 eyes, the PF was implanted in the right eye. Eleven of these 83 eyes (13.25%) had prior incisional glaucoma surgery (four eyes underwent TE; seven eyes underwent deep sclerectomy). Between the two groups, there was no statistically significant difference in age (*P* = 0.324), laterality of the operated eye (*P* = 0.545) and sex (*P* = 0.242).

**Table 1:** Baseline characteristics of patients undergoing trabeculectomy (TE) or PRESERFLO (PF) implantation

Parameter	TE	PF MicroShunt	*P*
Number of eyes (n)	104	83	n.a
Age (years)	70.00 ± 12.70	75.69 ± 10.84	0.324
Laterality (right/left)	51/53	37/46	0.545
Sex (female/male)	64/40	44/39	0.242
Ethnicity			
Caucasian/White	102	82	–
Hispanic	1	1	–
African American	1	0	–
Baseline IOP (mmHg)	24.09 ± 1.15	21.67 ± 0.77	0.160
Number of IOP-lowering medication	3.25 ± 0.14	3.51 ± 0.14	0.360
Number and types of prior incisional glaucoma surgeries	7 (7 × deep sclerectomy)	11 (4 × TE and 7 × deep sclerectomy)	0.133*
Number of phakic patients	65	57	0.380*
BCVA (logMAR)	0.33 ± 0.03	0.31 ± 0.04	0.547
Visual field mean defect (dB)	− 11.54 ± 0.93	− 11.17 ± 1.66	0.892
Mean peripapillary RFNL thickness (µm)	62.79 ± 1.94	62.62 ± 2.05	0.545
Medical glaucoma treatment prior to surgery			
Beta blockers	89	68	0.55*
Carboanhydrase inhibitors	85	74	0.22*
Alpha agonists	67	46	0.23*
Prostaglandins	85	71	0.56*
Systemic carboanhydrase inhibitors	22	28	0.12*

*IOP* = intraocular pressure; *BCVA* = best-corrected visual acuity; *RNFL* = retinal nerve fibre layer; *TE* = trabeculectomy; *PF* = PRESERFLO. Mann–Whitney U test was performed unless otherwise stated. *Fisher’s exact test

**Fig. 1:**
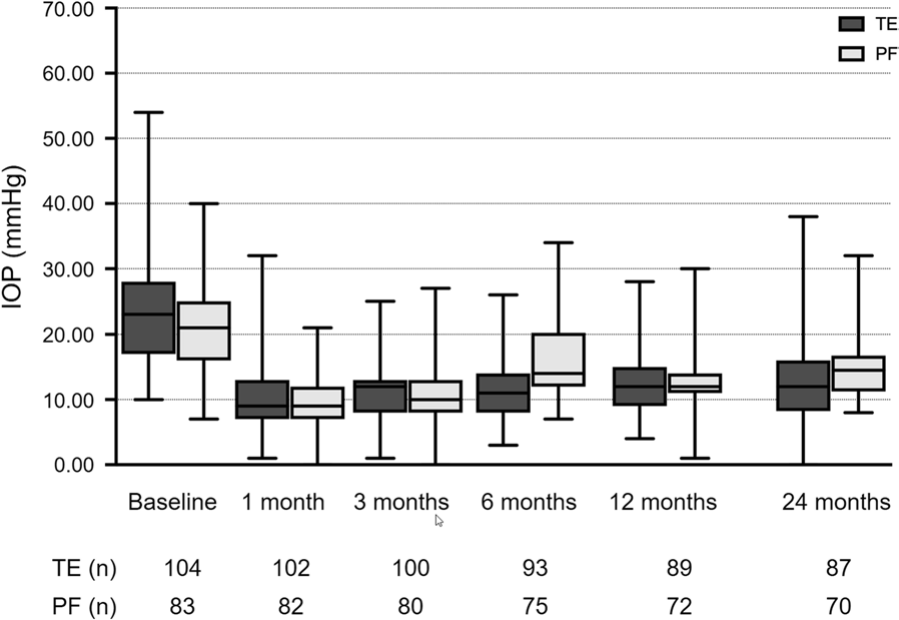
Mean intraocular pressure (IOP) development over the course of two years after TE or PF implantation. TE, trabeculectomy; PF, PRESERFLO

The mean course of IOP after TE or PF implantation is shown in Table 2 and Fig. 1. The mean IOP at baseline before surgery were 24.09 ± 1.15 mmHg and 21.67 ± 0.77 mmHg in the TE and PF implantation groups, respectively (*P* = 0.160). This decreased to 10.72 ± 0.82 mmHg (*P* < 0.001; compared to baseline) and 9.26 ± 0.40 mmHg (*P* < 0.001; compared to baseline) at the one-month follow-up after TE and PF implantation, respectively (TE *vs.* PF: *P* = 0.508). During the further postoperative course, the mean IOP increased to 12.18 ± 0.73 mmHg (*P* < 0.001) and 12.75 ± 0.75 mmHg (*P* < 0.001) at one year (TE *vs.* PF: *P* = 0.534) and 11.37 ± 1.13 mmHg (*P* < 0.001) and 15.50 ± 1.54 mmHg (*P* = 0.028) at two years after surgery (TE *vs.* PF: *P* = 0.183) in the TE and PF groups, respectively. This equates to a mean IOP decrease of 52.84% and 28.47% after two years in the TE and PF groups, respectively.

**Table 2:** Development of intraocular pressure (IOP), number of IOP-lowering medications and BCVA during 24 months of postoperative follow-up after trabeculectomy (TE) or PRESERFLO (PF) implantation

Parameter		TE	P*	PF MicroShunt	P*	*P*^*#*^
IOP (mmHg)	Baseline	24.09 ± 1.15 (22.50, 11.00)	n.a	21.67 ± 0.77 (21.00, 8.50)	n.a	0.160
	3 months	10.91 ± 0.66 (11.00, 5.00)	** < 0.001**	11.19 ± 0.78 (10.00, 4.50)	** < 0.001**	0.925
	6 months	11.94 ± 0.74 (11.00, 6.00)	** < 0.001**	15.97 ± 0.99 (14.00, 7.50)	** < 0.001**	** < 0.001**
	12 months	12.18 ± 0.73 (12.00, 6.00)	** < 0.001**	12.75 ± 0.75 (12.00, 3.00)	** < 0.001**	0.534
	24 months	11.37 ± 1.13 (12.00, 7.00)	** < 0.001**	15.50 ± 1.54 (14.50, 4.50)	**0.028**	0.183
Medication (n)	Baseline	3.25 ± 0.14 (4.00, 1.00)	n.a	3.51 ± 0.14 (4.00, 1.00)	n.a	0.360
	3 months	0.20 ± 0.10 (0.00 0.00)	** < 0.001**	0.12 ± 0.07 (0.00, 0.00)	** < 0.001**	0.687
	6 months	0.43 ± 0.14 (0.00, 0.00)	** < 0.001**	0.45 ± 0.16 (0.00, 0.00)	** < 0.001**	0.460
	12 months	0.60 ± 0.18 (0.00, 0.00)	** < 0.001**	0.64 ± 0.19 (0.00, 1.00)	** < 0.001**	0.558
	24 months	0.53 ± 0.14 (0.00 1.00)	** < 0.001**	1.06 ± 0.43 (0.00, 1.30)	** < 0.001**	0.589
BCVA (logMAR)	Baseline	0.33 ± 0.03 (0.20, 0.30)	n.a	0.31 ± 0.04 (0.20, 0.30)	n.a	0.547
	3 months	0.36 ± 0.04 (0.20, 0.40)	0.535	0.26 ± 0.04 (0.20, 0.30)	0.574	0.289
	6 months	0.28 ± 0.04 (0.20, 0.30)	0.172	0.28 ± 0.05 (0.20, 0.40)	0.898	0.944
	12 months	0.34 ± 0.04 (0.30, 0.40)	0.923	0.33 ± 0.07 (0.20, 0.20)	0.868	0.302
	24 months	0.38 ± 0.05 (0.20, 0.40)	0.223	0.40 ± 0.14 (0.20, 0.40)	0.628	0.709

Bold values indicate statistically significant

*IOP* = intraocular pressure; *BCVA* = best-corrected visual acuity; *TE* = trabeculectomy; *PF* = PRESERFLO

^*^ Wilcoxon-test; ^#^Mann–Whitney U test

**Fig. 2:**
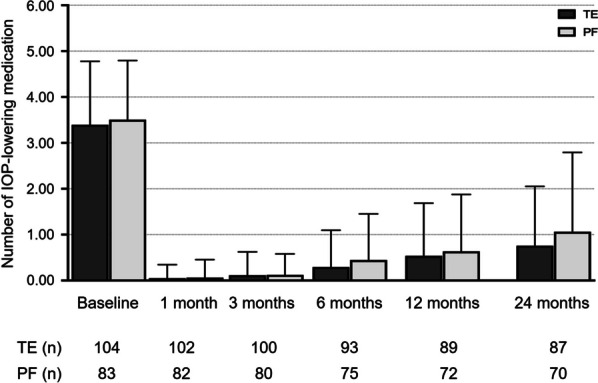
Development of the mean number of necessary IOP-lowering medication over the course of two years after TE or PF implantation. IOP, intraocular pressure; TE, trabeculectomy; PF, PRESERFLO

**Fig. 3:**
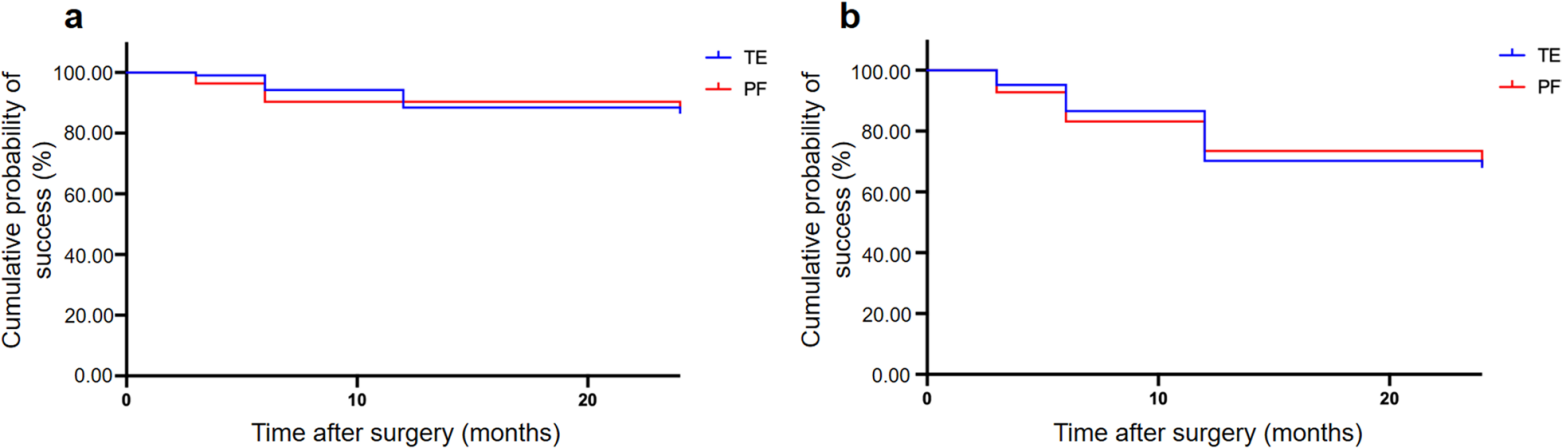
Kaplan–Meier plots showing rates for (**a**) qualified and (**b**) complete success in the TE and PF groups during 24 months of postsurgical follow-up; TE, trabeculectomy; PF, PRESERFLO

The number of IOP-lowering medications in the two groups followed a similar course as IOP during the two years of postsurgical follow-up (Table 2 and Fig. 2). The mean number of IOP-lowering medications at baseline was 3.25 ± 0.14 and 3.51 ± 0.14 (*P* = 0.360) in the TE and PF implantation groups, respectively. These numbers decreased to 0.43 ± 0.14 (*P* < 0.001), 0.60 ± 0.18 (*P* < 0.001), and 0.53 ± 0.14 (*P* < 0.001) at 6, 12 and 24 months after surgery in the TE group, and to 0.45 ± 0.16 (*P* < 0.001), 0.64 ± 0.19 (*P* < 0.001), and 1.06 ± 0.43 (*P* < 0.001) at 6, 12, and 24 months after surgery in the PF implantation group. IOP and number of IOP lowering agents applied led to a qualified success rate of 90% and 92% and a complete success rate of 69% and 72% after two years in the TE and PF groups, respectively. Further results concerning success rates are depicted as Kaplan–Meier plots in Fig. 3.

**Fig. 4:**
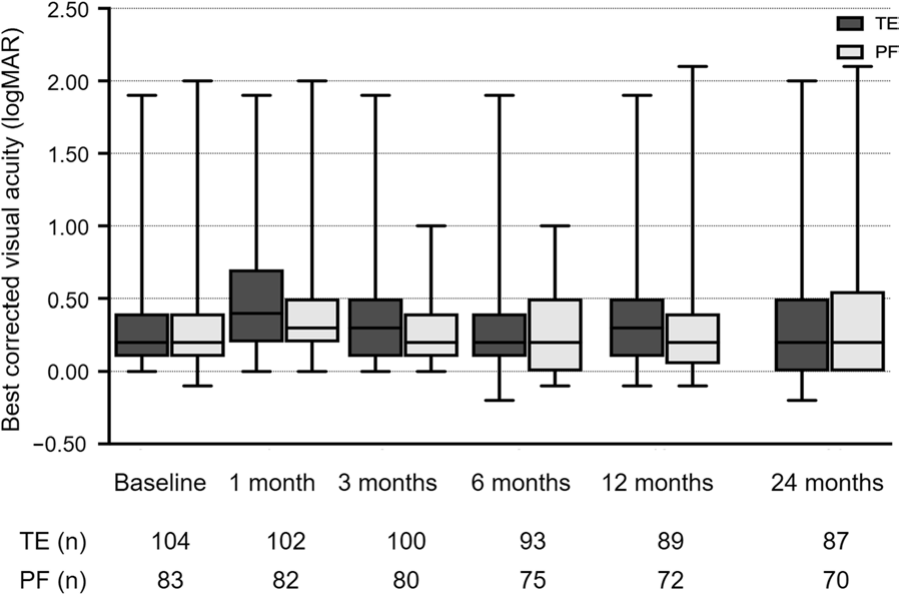
Course of the mean BCVA over the two years of follow-up after TE or PF implantation. BCVA, best-corrected visual acuity; TE, trabeculectomy; PF PRESERFLO

The mean BCVA at baseline was 0.33 ± 0.03 logMAR and 0.31 ± 0.04 logMAR (*P* = 0.547) for the TE and PF implantation groups, respectively (Table 2 and Fig. 4). As expected, mean BCVA decreased significantly in both treatment groups at the one-day and one-month follow-up visits (TE: *P* < 0.001 at one day, *P* < 0.001 at one month; PF: *P* < 0.001 at one day, *P* < 0.001 at one month). BCVA then returned to preoperative values at the three-month follow-up visit and remained stable through the two years of postsurgical follow-up, with a mean BCVA of 0.38 ± 0.05 logMAR (*P* = 0.223) and 0.40 ± 0.14 logMAR (*P* = 0.628) in the TE and PF implantation groups, respectively (TE *vs.* PF: *P* = 0.709).

**Fig. 5:**
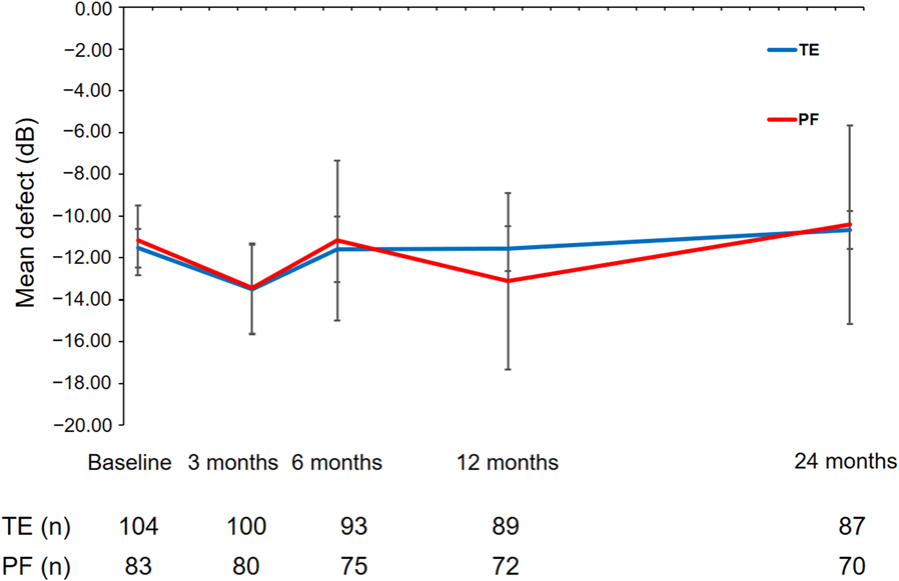
Course of the visual field mean deviation over the two years of follow-up after TE or PF implantation. TE, trabeculectomy; PF, PRESERFLO

At baseline, MD of visual field was − 11.54 ± 0.93 dB and − 11.17 ± 1.66 dB in the TE and PF implantation groups, respectively (*P* = 0.892) (Table 3 and Fig. 5). In the TE group, MD remained stable during follow-up with − 11.57 ± 1.07 dB (*P* = 0.147) and − 10.67 ± 0.91 dB (*P* = 0.226) at one and two years after surgery, respectively. In the PF implantation group, MD increased to − 13.12 ± 4.20 dB (*P* = 0.868) at one year but decreased to − 10.40 ± 4.75 dB (*P* = 0.628) at two years after surgery, which was not statistically significant compared to baseline values. Similarly, statistical analysis comparing MD in the TE and PF implantation groups showed no statistically significant difference for the results at six months (*P* = 0.859), one year (*P* = 0.914), and two years (*P* = 0.123) after surgery.

**Table 3:** Development of mean defect of standard automated perimetry (SAP) and mean peripapillary retinal nerve fibre layer (RNFL) thickness during 24 months of postoperative follow-up after trabeculectomy (TE) or PRESERFLO (PF) implantation

Parameter		TE	P*	PF MicroShunt	P*	*P*^*#*^
SAP mean defect (dB)	Baseline	− 11.54 ± 0.93 (10.50, 9.70)	n.a	− 11.17 ± 1.66 (10.20, 6.60)	n.a	0.892
	6 months	− 11.60 ± 1.56 (11.50, 9.50)	0.407	− 11.16 ± 3.82 (10.20, 5.50)	0.180	0.859
	12 months	− 11.57 ± 1.07 (12.60, 9.00)	0.147	− 13.12 ± 4.20 (11.30, 2.60)	0.868	0.914
	24 months	− 10.67 ± 0.91 (11.30, 10.60)	0.226	− 10.40 ± 4.75 (10.80, 3.80)	0.628	0.123
RNFL thickness (µm)	Baseline	62.79 ± 1.94 (58.50, 26.00)	n.a	62.62 ± 2.05 (57.00, 22.80)	n.a	0.545
	3 months	61.27 ± 3.05 (58.00, 26.00)	0.174	60.99 ± 2.02 (57.00, 24.00)	0.079	0.892
	6 months	58.67 ± 2.75 (54.00, 25.00)	** < 0.001**	60.36 ± 2.00 (57.00, 23.60)	0.275	0.344
	12 months	58.97 ± 2.39 (57.00, 27.50)	** < 0.001**	60.04 ± 2.10 (60.00, 23.50)	0.857	0.584
	24 months	57.41 ± 1.81 (54.00, 27.00)	** < 0.001**	60.22 ± 1.98 (60.00, 22.80)	0.182	0.453

Bold values indicate statistically significant

*SAP* = standard automated perimetry; *RNFL* = retinal nerve fibre layer; *TE* = trabeculectomy; *PF* = PRESERFLO

^*^Wilcoxon-test; ^#^Mann–Whitney U test

**Fig. 6:**
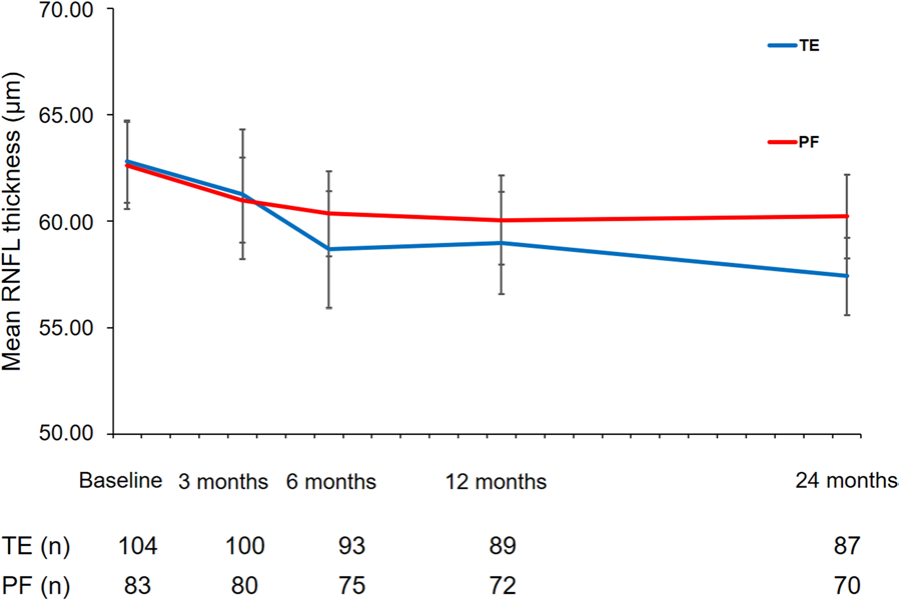
Course of the mean RNFL thickness measured using peripapillary OCT scan over the two years of follow-up after TE or PF implantation. RNFL, retinal nerve fibre layer; OCT, optical coherence tomography; TE, trabeculectomy; PF, PRESERFLO

Mean circumpapillary RNFL thickness at baseline was 62.79 ± 1.94 µm and 62.62 ± 2.05 µm (*P* = 0.545) in the TE and PF implantation groups, respectively (Table 3 and Fig. 6). During the follow-up, RNFL thickness decreased steadily in both treatment groups. In the TE group, mean RNFL thickness was 58.97 ± 2.39 µm (*P* < 0.001) and 57.41 ± 1.81 µm (*P* < 0.001) at one and two years after surgery, respectively. This represents a loss of 6.08% and 8.57% of mean RNFL thickness compared to mean baseline values. In the PF implantation group, mean RNFL thickness decreased to 60.04 ± 2.10 µm (*P* = 0.857) and 60.22 ± 1.98 µm (*P* = 0.182) at one and two years after surgery, respectively, representing a loss of 3.83% during two years of postoperative follow-up. RNFL loss continued steadily until the one-year postoperative follow-up in both treatment groups. However, there were no statistically significant differences between the TE and PF implantation groups at six months (*P* = 0.344), one year (*P* = 0.584), or two years (*P* = 0.453) after surgery concerning RNFL loss.

Complications noted during postsurgical follow-up were comparable in TE and PF groups and further statistical analysis did not reveal differences of statistical significance between both groups (Table 4). During the 24-month follow-up after surgery several (surgical) interventions were performed in the TE and PF groups (Table 5). During follow-up, the number of eyes receiving subconjunctival injections of 5-FU and the number of eyes undergoing a needling procedure under the operating microscope was larger in the PF group. Further statistical analysis revealed the different amounts of 5-FU used and performance of needlings are of statistical significance (Table 5).

**Table 4:** Frequency of postsurgical complications after trabeculectomy (TE) and PRESERFLO (PF) during 24 months of postoperative follow-up

Parameters	TE	PF MicroShunt	P*
Early (*<* 3 months after surgery)			
Hyphema	12	11	0.82
Shallow anterior chamber	10	7	1.00
Choroidal effusion	16	17	0.44
Suprachoroidal haemorrhage	0	0	1.00
Aqueous misdirection syndrome	2	0	0.50
Corneal dellen	0	0	1.00
Corneal erosion	12	7	0.63
Late (*>* 3 months after surgery)			
Encapsulated bleb	10	12	0.36
Hypotonia maculopathy	3	1	0.63
Macular oedema	2	1	1.00
Endophthalmitis	0	0	1.00
Tube retraction	0	0	1.00

*TE* = trabeculectomy; *PF* = PRESERFLO

^*^ Fisher’s exact test

**Table 5:** Frequency of further (surgical) interventions and total number of appointments during 24 months of postoperative follow-up after trabeculectomy (TE) or PRESERFLO (PF) implantation

Parameter	TE	PF MicroShunt	P*
Number of 5-FU injections	82	186	** < 0.01**
Number of eyes receiving 5-FU injections (total/percentage)	25 (24.04%)	52 (62.65%)	** < 0.01**
Number of needlings	22	32	**0.01**
Number of eyes receiving needlings (total/percentage)	19 (18.27%)	28 (33.74%)	**0.02**
AC reformation	7 (6.73%)	4 (4.82%)	0.76
Wound suture/bleb leakage	2 (1.92%)	2 (2.41%)	1.00
Laser suture lysis	13 (12.50%)	0	** < 0.01**
Releasable suture removal	1 (0.96%)	0	1.00
Revision surgery	6	8	0.41
Cataract surgery (phacoemulsification)	6	2	0.30
Secondary glaucoma surgery	4 (1 × Baerveldt tube, 1 × Ahmed tube, 2 × Cyclophotocoagulation)	4 (3xTE, 1 × Baerveldt tube)	1.00
Mean number of appointments per case	10.10 ± 3.60	9.40 ± 2.80	0.38

Bold values indicate statistically significant

*TE* = trabeculectomy; *PF* = PRESERFLO; *5-FU* = 5-fluorouracil

^*^Fisher’s exact test

In the TE group, 12 eyes were in an early disease stage (MD < 5.00 dB), 41 in a moderate (MD 5.00–12.00 dB) and 51 in an advanced disease stage (MD > 12.00 dB). Accordingly, in the PF group, nine eyes were in an early, 31 eyes in a moderate and 43 eyes in an advanced disease stage. In the TE group, mean baseline IOP was 24.67 ± 2.65 mmHg, 21.87 ± 1.26 mmHg and 25.46 ± 1.37 mmHg before surgery, which dropped to 11.92 ± 1.58 mmHg, 12.38 ± 0.99 mmHg and 12.18 ± 0.69 mmHg after one year and 11.92 ± 1.25 mmHg, 13.19 ± 1.2 and 12.37 ± 1.04 mmHg after two years in the subgroups of early, moderate, and advanced disease stages. Further statistical analysis of the different IOP results in the three subgroups did not reveal statistical significance (Kruskal–Wallis test; for results see Table 6). In the PF group, mean IOP dropped from mean baseline values of 20.67 ± 1.50 mmHg, 22.97 ± 1.48 mmHg and 20.95 ± 0.99 mmHg in the subgroups of early, moderate, and advanced disease stages to 12.00 ± 1.34 mmHg, 13.04 ± 1.06 mmHg and 12.95 ± 1.08 mmHg after one year and 15.00 ± 1.30 mmHg, 16.50 ± 1.44 mmHg and 15.18 ± 1.37 mmHg after two years, respectively. Further statistical analysis in the TE group did not reveal statistical significance for the differences between the three subgroups (Table 6). As for IOP, no differences of statistical significance could be found when comparing results for mean number of medications and achieved complete and qualified success levels as described above in the three subgroups of different disease stages in the TE and PF groups at baseline as well as 12 and 24 months after surgery.

**Table 6:** *P* values for comparisons between results for intraocular pressure (IOP), number of applied IOP-lowering medication and success levels at baseline (where applicable), 12 and 24 months after surgery between subgroups of early, moderate, and advanced disease stages in the trabeculectomy (TE) and PRESERFLO (PF) MicroShunt groups using Kruskal–Wallis test

Parameter	TE			PF MicroShunt		
	Baseline	12 months	24 months	Baseline	12 months	24 months
IOP	0.27	0.41	0.84	0.66	0.74	0.92
Medication	0.18	0.98	0.56	0.23	0.35	0.58
Complete success	n.a	0.87	0.74	n.a	0.47	0.37
Qualified success	n.a	0.46	0.31	n.a	0.33	0.50

*TE* = trabeculectomy; *PF* = PRESERFLO; *IOP* = intraocular pressure

## Discussion

With this study, we demonstrate that IOP and the number of IOP-lowering medications can be lowered by PF implantation with a comparable efficacy to TE over a two-year intermediate follow-up in POAG eyes. However, despite the reduction of IOP and treatment burden of IOP lowering medication, the decrease in peripapillary RNFL thickness continues after surgery, and was more pronounced in our TE group. This suggests that there is continuing RNFL decline even after IOP normalization for several months after surgery. After the 12-month follow-up, RFNL thickness remained stable in both TE and PF implantation groups.

TE was first described in its current form in the 1960s and has long been considered the gold standard in incisional glaucoma surgery [30, 31]. Several studies with large numbers of eyes and long follow-up periods underline the pronounced and substantial efficacy of this technique. In a nationwide multicenter survey conducted in the 1990s across the UK, including over 1200 eyes, the mean IOP was reduced from 26.2 to 14.4 mmHg one year after surgery. In addition, IOP decreased by more than one third compared to baseline in 66.6% of cases without additional application of IOP-lowering medication [32]. In another multicenter clinical trial of 428 POAG eyes, mean IOP was significantly reduced from 23.0 ± 5.5 mmHg to 12.4 ± 4.0 mmHg, while the mean number of IOP-lowering medications was reduced from 2.5 ± 0.9 to 0.1 ± 0.4 at 24 months of follow-up [12].

The PF MicroShunt is implanted using an ab externo approach and was designed to bypass the physiological outflow pathway of aqueous humour via trabecular meshwork and Schlemm’s canal. The PF implant directs the aqueous humour under conjunctiva and Tenon’s capsule, partially imitating the mechanism of TE. A large multicenter trial confirmed the efficacy of the PF device in lowering IOP and reducing the use of IOP-lowering medications over a follow-up period of 24 months. The mean IOP decreased from 21.7 ± 3.4 mmHg to 14.5 ± 4.6 mmHg and the mean number of IOP-lowering medications decreased from 2.1 ± 1.3 to 0.5 ± 0.9 during follow-up [21]. The number and severity of ocular adverse events were reported to be low. In another single-center study comparing the postoperative outcomes after either TE and PF MicroShunt implantation in a cohort of 52 POAG eyes, demonstrated that mean value and fluctuation of diurnal IOP and the number of IOP-lowering medications at the six-month follow-up visit were reduced compared to baseline in both groups and the differences were of statistical significance [33]. For the comparison between two groups, there was no statistically significant difference in the above outcomes. The mean diurnal IOP decreased from 15.9 mmHg (rang: 13.8–26.5 mmHg) to 10.8 mmHg (rang: 9.5–12.2 mmHg) in the TE group and from 17.1 mmHg (rang: 13.3–20.4 mmHg) to 10.3 mmHg (rang: 7.6–11.8 mmHg) in the PF group, while the mean number of IOP-lowering medications decreased from 4 (rang: 3–5) to 0 (rang: 0–0) and from 4 (rang: 3–4) to 0 (rang: 0–0) in the TE and PF groups, respectively.

Baker and colleagues performed a randomized multicenter trial comparing the results after TE and PF MicroShunt implantation in a comparable group of POAG cases and found that IOP results were lower (11.1 ± 4.3 mmHg vs. 14.3 ± 4.9 mmHg in the TE and PF groups, respectively) and success rates were larger in the TE group (72.7% vs. 53.9%) [34]. A direct comparison to the presented results must be made with caution, because of the different study designs, different inclusion criteria (previous glaucoma surgery allowed in the hereby presented study), different baseline IOP values and different study ethnicities, amongst others. Apart from this, it might have been that target IOP values in our population undergoing TE were not as low and threshold for performing secondary interventions (5-FU application, needling procedures, revision surgery) was lower in our study population. 62.7% of eyes undergoing PF MicroShunt implantation received 5-FU injections and 33.7% of PF cases underwent a needling procedure. However, given the high rate of performed secondary interventions and the comparable number of occurring adverse events and complications after surgery benefits of PF MicroShunt implantation over TE are debatable.

Although effective in lowering IOP and the number of glaucoma medications, TE can be associated with various complications ranging from mild discomfort to complete loss of visual function and organ loss, and is often associated with relatively long recovery times [35, 36]. This has led to the desire to develop less invasive surgical techniques such as the PF implantation which so far have shown promising results in the short to medium term [37]. With the results of this study, we showed that decrease of IOP and IOP-lowering medication is comparable between TE and PF over a postsurgical follow-up of two years. However, we also demonstrated that further postsurgical wound modulation was more often necessary in the PF group. Total number and percentage of eyes receiving subconjunctival 5-FU injections and needlings was larger in the PF than in the TE group.

In the presented results of the PF group, we found an increase of mean IOP between the three- and six-month follow-up examination from 11.19 ± 0.78 mmHg to 15.97 ± 0.99 mmHg which decreased again to 12.75 ± 0.75 mmHg between the six- and 12-month follow-up examination. Further analysis of the collected data showed that 28 of the 32 performed needling procedures were performed between six and 12 months after PF MicroShunt implantation and may be the reason leading to the following decrease in mean IOP at the 12-month examination. In the TE group, only 12 of the 22 needling procedures fell into this time between six and 12 months after surgery. This should be kept in mind when counselling patients after PF MicroShunt implantation. However, the different rates for necessary 5-FU applications, performed needling procedures and other necessary further interventions did not lead to markedly higher number of appointments of patients undergoing TE or PF MicroShunt implantation at our outpatient department. The spike of mean IOP in the PF group at the six months follow-up examination might also be related to this.

IOP lowering is often described as the primary goal of medical and surgical interventions in glaucoma. However, preservation of retinal ganglion cells from apoptotic cell death should be the underlying main goal of glaucoma treatment. Several studies have shown that RGC loss continues after glaucoma surgery. In another study of POAG eyes that underwent TE or XEN microstent implantation, we already demonstrated that although the preoperative target IOP levels were achieved, the peripapillary RNFL thickness continued to decrease during the first year after surgery and remained stable during the second year after surgery [38]. This further RNFL loss occurred without evidence of further functional deterioration and was more pronounced in the TE group than in the XEN implantation group. This further loss of RNFL thickness during postoperative follow-up, while visual field test results remained stable, was comparable to the results of the current study. Similarly, Chua et al. reported a further postoperative decrease in peripapillary RNFL thickness of 4.21 µm during the first year after TE in 130 eyes [39]. In this study, the decrease in peripapillary RNFL thickness in the first year after surgery was − 3.80 µm and − 2.60 µm in the TE and PF implantation groups, respectively.

However, it has also been demonstrated previously that the rate of RGC loss slows after surgical intervention and IOP control. Demirtas et al. [40] presented data from 32 POAG eyes that underwent TE in which the mean peripapillary RNFL thickness decreased significantly by − 9.62 ± 14.39 µm in the first year after surgery (*P* = 0.002) and remained stable in the second and third year after surgery. With this knowledge about the expected further course of RNFL changes after surgery and the routine problems with patient adherence and persistence to medical glaucoma treatment, it might be advisable to opt for surgical glaucoma intervention earlier in the disease course. However, prior to this more refined analysis and patient evaluation, modules are urgently needed to identify individuals in need of more aggressive glaucoma treatment to prevent overtreatment.

This study has several limitations. Firstly, it would be desirable to include a larger number of eyes and to follow them for a longer period, especially regarding occurring further changes of visual field results and RNFL thickness. Secondly, for future analysis, addition of further aspects (e.g., IOP fluctuation, peak IOP, axial length, anterior chamber depth) and analysis of their impact on IOP and surgical success would be worthwhile. Finally, it would be of interest in eyes treated with PF implantation to use different concentrations of MMC during surgery and compare IOP and medication results between these groups during follow-up as some evidence exists for higher MMC concentrations (0.4 or 0.5 mg/mL) leading to more favourable postoperative results [41]. Application of higher MMC doses should be safe as the resulting filtration zone after PRESERFLO MicroShunt implantation is located further posterior from the corneal limbus compared to TE. Therefore, higher doses probably should lead to lower postoperative IOP results maybe also equalizing the hereby presented differences for results and necessary interventions between TE and PRESERFLO MicroShunt implantation groups and evening out the pressure spike found in our six months results of the PF group. Nevertheless, a strength of our analysis is the inclusion of functional (visual fields) and morphological outcomes (peripapillary OCT scans) measured regularly during follow-up. Future studies may also utilize more refined analysis settings that consider the inner retinal morphology of the macular region.

## Conclusion

This study provides two-year follow-up data on the outcomes of patients who underwent PF MicroShunt implantation compared to TE. We demonstrated the medium-term efficacy of PF implantation in reducing IOP and the number of IOP-lowering medications in patients with POAG. This study also provides evidence that structural glaucomatous damage may continue while the functional glaucomatous damage remains stable after glaucoma surgery. PF showed potential to stabilize functional and structural glaucomatous damage. Future studies are needed to monitor the long-term efficacy of PF in both IOP-lowering and stabilizing glaucomatous progression.

## Data Availability

The datasets used and/or analysed during the current study are available from the corresponding author on reasonable request.

## Abbreviations

PF: PRESERFLO
TE: Trabeculectomy
POAG: Primary open-angle glaucoma
RGC: Retinal ganglion cell
IOP: Intraocular pressure
RNFL: Retinal nerve fiber layer
MIGS: Minimally invasive glaucoma surgery
OCT: Optical coherence tomography
VF: Visual field
MD: Mean defect
BCVA: Best-corrected visual acuity
MMC: Mitomycin C
BSS: Balanced salt solution

## References

[1] Jonas JB, Aung T, Bourne RR, Bron AM, Ritch R, Panda-Jonas S (2017). Glaucoma. Lancet.

[2] Quigley HA (1995). Ganglion cell death in glaucoma: pathology recapitulates ontogeny. Aust N Z J Ophthalmol.

[3] Levkovitch-Verbin H (2015). Retinal ganglion cell apoptotic pathway in glaucoma: initiating and downstream mechanisms. Prog Brain Res.

[4] Leske MC, Wu SY, Hennis A, Honkanen R, Nemesure B, BESs Study Group (2008). Risk factors for incident open-angle glaucoma: the Barbados Eye Studies. Ophthalmology.

[5] Weinreb RN, Aung T, Medeiros FA (2014). The pathophysiology and treatment of glaucoma: a review. JAMA.

[6] Weinreb RN, Khaw PT (2004). Primary open-angle glaucoma. Lancet.

[7] Heijl A, Leske MC, Bengtsson B, Hyman L, Bengtsson B, Hussein M (2002). Reduction of intraocular pressure and glaucoma progression: results from the Early Manifest Glaucoma Trial. Arch Ophthalmol.

[8] Leske MC, Heijl A, Hussein M, Bengtsson B, Hyman L, Komaroff E (2003). Factors for glaucoma progression and the effect of treatment: the Early Manifest Glaucoma Trial. Arch Ophthalmol.

[9] Casson RJ (2022). Medical therapy for glaucoma: a review. Clin Exp Ophthalmol.

[10] Schmidl D, Schmetterer L, Garhöfer G, Popa-Cherecheanu A (2015). Pharmacotherapy of glaucoma. J Ocul Pharmacol Ther.

[11] Razeghinejad MR, Fudemberg SJ, Spaeth GL (2012). The changing conceptual basis of trabeculectomy: a review of past and current surgical techniques. Surv Ophthalmol.

[12] Kirwan JF, Lockwood AJ, Shah P, Macleod A, Broadway DC, King AJ (2013). Trabeculectomy in the 21st century: a multicenter analysis. Ophthalmology.

[13] Edmunds B, Thompson JR, Salmon JF, Wormald RP (2001). The National Survey of Trabeculectomy. II. Variations in operative technique and outcome. Eye (Lond).

[14] Landers J, Martin K, Sarkies N, Bourne R, Watson P (2012). A twenty-year follow-up study of trabeculectomy: risk factors and outcomes. Ophthalmology.

[15] Rulli E, Biagioli E, Riva I, Gambirasio G, De Simone I, Floriani I (2013). Efficacy and safety of trabeculectomy vs nonpenetrating surgical procedures: a systematic review and meta-analysis. JAMA Ophthalmol.

[16] Zahid S, Musch DC, Niziol LM, Lichter PR (2013). Risk of endophthalmitis and other long-term complications of trabeculectomy in the Collaborative Initial Glaucoma Treatment Study (CIGTS). Am J Ophthalmol..

[17] Kim EA, Law SK, Coleman AL, Nouri-Mahdavi K, Giaconi JA, Yu F (2015). Long-term bleb-related infections after trabeculectomy: incidence, risk factors, and influence of bleb revision. Am J Ophthalmol.

[18] Birnbaum FA, Neeson C, Solá-Del VD (2021). Microinvasive glaucoma surgery: an evidence-based review. Semin Ophthalmol.

[19] Lavia C, Dallorto L, Maule M, Ceccarelli M, Fea AM (2017). Minimally-invasive glaucoma surgeries (MIGS) for open angle glaucoma: a systematic review and meta-analysis. PLoS ONE.

[20] Pinchuk L, Riss I, Batlle JF, Kato YP, Martin JB, Arrieta E (2017). The development of a micro-shunt made from poly(styrene-block-isobutylene-block-styrene) to treat glaucoma. J Biomed Mater Res B Appl Biomater.

[21] Beckers HJM, Aptel F, Webers CAB, Bluwol E, Martínez-de-la-Casa JM, García-Feijoó J (2022). Safety and effectiveness of the PRESERFLO® MicroShunt in primary open-angle glaucoma: results from a 2-year multicenter study. Ophthalmol Glaucoma.

[22] Seuthe AM, Erokhina M, Szurman P, Haus A (2023). One year results of PRESERFLO® MicroShunt implantation for refractory glaucoma. J Glaucoma.

[23] Van Lancker L, Saravanan A, Abu-Bakra M, Reid K, Quijano C, Goyal S (2023). Clinical outcomes and cost analysis of PreserFlo versus trabeculectomy for glaucoma management in the United Kingdom. Ophthalmol Glaucoma.

[24] Ibarz Barberá M, Martínez-Galdón F, Caballero-Magro E, Rodríguez-Piñero M, Tañá-Rivero P (2022). Efficacy and safety of the Preserflo Microshunt with mitomycin C for the treatment of open angle glaucoma. J Glaucoma.

[25] Tanner A, Haddad F, Fajardo-Sanchez J, Nguyen E, Thong KX, Ah-Moye S (2023). One-year surgical outcomes of the PreserFlo MicroShunt in glaucoma: a multicentre analysis. Br J Ophthalmol.

[26] Bhayani R, Martínez de la Casa JM, Figus M, Klabe K, Rabiolo A, Mercieca K (2023). Short-term safety and efficacy of Preserflo™ Microshunt in glaucoma patients: a multicentre retrospective cohort study. Eye (Lond).

[27] Gambini G, Carlà MM, Giannuzzi F, Caporossi T, De Vico U, Savastano A (2022). PreserFlo® MicroShunt: an overview of this minimally invasive device for open-angle glaucoma. Vision (Basel).

[28] Khaw PT, Chiang M, Shah P, Sii F, Lockwood A, Khalili A (2017). Enhanced trabeculectomy: the moorfields safer surgery system. Glaucoma Surg.

[29] Pinchuk L, Riss I, Batlle JF, Beckers H, Stalmans I. An ab externo minimally invasive aqueous shunt comprised of a novel biomaterial. Current Developments in Glaucoma Surgery and Migs: Kugler Publications; 2020. p. 181–92.

[30] Cairns JE (1968). Trabeculectomy. Preliminary report of a new method. Am J Ophthalmol..

[31] Stalmans I, Gillis A, Lafaut AS, Zeyen T (2006). Safe trabeculectomy technique: long term outcome. Br J Ophthalmol.

[32] Edmunds B, Thompson JR, Salmon JF, Wormald RP (2002). The National Survey of Trabeculectomy. III. Early and late complications. Eye (Lond).

[33] Pillunat KR, Herber R, Haase MA, Jamke M, Jasper CS, Pillunat LE (2022). PRESERFLO™ MicroShunt versus trabeculectomy: first results on efficacy and safety. Acta Ophthalmol.

[34] Baker ND, Barnebey HS, Moster MR, Stiles MC, Vold SD, Khatana AK (2021). Ab-externo MicroShunt versus trabeculectomy in primary open-angle glaucoma: one-year results from a 2-year randomized, multicenter study. Ophthalmology.

[35] Gedde SJ, Feuer WJ, Lim KS, Barton K, Goyal S, Ahmed IIK (2020). Treatment outcomes in the Primary Tube versus trabeculectomy study after 3 years of follow-up. Ophthalmology.

[36] Fontana H, Nouri-Mahdavi K, Lumba J, Ralli M, Caprioli J (2006). Trabeculectomy with mitomycin C: outcomes and risk factors for failure in phakic open-angle glaucoma. Ophthalmology.

[37] Saheb H, Ahmed II (2012). Micro-invasive glaucoma surgery: current perspectives and future directions. Curr Opin Ophthalmol.

[38] Bormann C, Busch C, Rehak M, Schmidt M, Scharenberg C, Ziemssen F (2022). Two year functional and structural changes-a comparison between trabeculectomy and XEN microstent implantation using spectral domain optical coherence tomography. J Clin Med.

[39] Chua J, Kadziauskienė A, Wong D, Ašoklis R, Lesinskas E, Quang ND (2020). One year structural and functional glaucoma progression after trabeculectomy. Sci Rep.

[40] Demirtaş AA, Karahan M, Erdem S, Aslan Kaya A, Keklikçi U (2021). Long-term effects of trabeculectomy in primary open-angle glaucoma on segmented macular ganglion cell complex alterations. Int Ophthalmol.

[41] Schlenker MB, Durr GM, Michaelov E, Ahmed IIK (2020). Intermediate outcomes of a novel standalone ab externo SIBS microshunt with mitomycin C. Am J Ophthalmol.

